# Curcumin Solubility and Bioactivity Enhancement Through Amorphization with Tryptophan via Supercritical Fluid Technology

**DOI:** 10.3390/ijms26020855

**Published:** 2025-01-20

**Authors:** Ewa Garbiec, Natalia Rosiak, Szymon Sip, Przemysław Zalewski, Judyta Cielecka-Piontek

**Affiliations:** Department of Pharmacognosy and Biomaterials, Poznan University of Medical Sciences, Rokietnicka 3 Str., 60-806 Poznan, Poland; ewa.garbiec@student.ump.edu.pl (E.G.); nrosiak@ump.edu.pl (N.R.); szymonsip@ump.edu.pl (S.S.); pzalewski@ump.edu.pl (P.Z.)

**Keywords:** curcumin, amorphous, tryptophan, supercritical fluid technology

## Abstract

Curcumin, a compound known for its antioxidant and neuroprotective properties, faces challenges due to its low water solubility, which can limit its effectiveness. One effective method to address this issue is through amorphization. Incorporating curcumin into a polymeric matrix to form amorphous solid dispersions is a common approach. Another strategy involves co-amorphous systems, where low-molecular-weight components act as co-formers. A recent innovative approach combines these strategies. This study used tryptophan as a co-former and prepared systems using supercritical fluid technology. The amorphous nature of two systems was confirmed through X-ray powder diffraction: one with 10% curcumin and a polymer, and another with 10% curcumin, a polymer, and tryptophan. Fourier-transform infrared analysis demonstrated molecular interactions among all components in the systems. Scanning electron microscopy revealed that the amorphization process significantly modified the morphology of the powder particles. The ternary system with tryptophan notably increased curcumin solubility by over 300-fold. The amorphous form of curcumin in both systems exhibited significantly higher dissolution rates compared to its crystalline form. The system with tryptophan showed more than a threefold improvement in permeability according to the PAMPA test. The enhanced solubility led to over a sixfold increase in antioxidant activity and a 25-fold improvement in the inhibition of the enzyme butyrylcholinesterase.

## 1. Introduction

Curcumin (CUR) is a bioactive compound found in turmeric, derived from the plant *Curcuma longa* [1]. Its many biological properties and therapeutic potential have been thoroughly investigated. Owing to its antibacterial, anti-inflammatory, and antioxidant properties, CUR has been researched for a variety of uses, including wound healing [2], rheumatoid arthritis treatment [3], the prevention of cardiovascular diseases [4], and as a potential treatment for neurodegenerative diseases [5,6] and depression [7,8,9].

CUR’s properties are ascribed to its polyphenolic structure, which allows it to demonstrate noteworthy biological activity. Despite its promising potential, CUR faces limitations due to its poor solubility, prompting research aimed at increasing its solubility to enhance its efficacy. Among others, many studies have investigated the usage of cyclodextrins as complexing agents. It has been shown that γ-cyclodextrin, hydroxypropyl-β-cyclodextrin (HPβCD), and β-cyclodextrin greatly improve CUR’s water solubility, stability, dissolution rate, and bioavailability [10,11,12,13,14]. Also, the application of various nanoparticles, such as those based on polymers, chitosan, starch, and alginate-polysorbate, has demonstrated promise in improving CUR’s solubility [15,16,17,18]. Another approach involves obtaining co-crystals. It has been shown that CUR’s solubility can be increased by co-crystallizing it with specific co-formers, such as salicylic acid, hydroxyquinol, cinnamic acid, resorcinol, pyrogallol, nicotinamide, resveratrol, and ascorbic acid [19,20,21,22,23,24].

Another effective method for enhancing solubility involves the conversion of a crystalline substance into its amorphous state. One explanation for the higher solubility of amorphous forms is that they have a higher free-energy state, which makes them more soluble than crystalline forms [25]. However, this solubility advantage could be compromised by the quick return to the crystalline state [26].

One notable advancement in addressing this issue has been the creation of amorphous solid dispersions (ASD), where the drug is dispersed at a molecular level within an amorphous polymer. The elevated glass transition temperature (Tg) resulting from the employment of high-Tg carrier polymers leads to a reduction in molecular mobility, thereby contributing to the heightened physical stability of these amorphous systems. Moreover, the molecular interactions between the drug and polymer often play a role in enhancing the stability [27]. Stable ASDs of CUR with enhanced solubility have been successfully achieved through various methods, such as solvent evaporation [28,29,30], spray-drying [31], rotary evaporation followed by cryomilling [32], and hot-melt extrusion [33].

An alternative method includes creating co-amorphous systems (CAMs) through the co-amorphization of low-molecular-weight compounds, such as amino acids, to establish intermolecular interactions that enhance the stability of the amorphous structure [34,35]. The literature has documented instances of co-amorphous systems of CUR prepared with folic acid dihydrate through liquid-assisted grinding [36], with artemisinin using the rotavaporized method [37], and with piperine via melting and the quench cooling method [38].

Techniques aimed at improving solubility by reducing crystallinity and particle size are gaining increasing attention, particularly those based on supercritical fluid (SCF) technology using supercritical carbon dioxide (scCO_2_) as an antisolvent. The CO_2_ utilized is non-flammable, chemically inert, and characterized by low critical parameters (31.1 °C, 7.38 MPa), together with high diffusivity and volatility, which simplify its removal from the product post-process [39]. The supercritical antisolvent (SAS) process and its modified variant, solution-enhanced dispersion by scCO_2_ (SEDS), have both been successfully employed to improve the properties of CUR. These methods reduce particle size and crystallinity, leading to an increased surface area. As a result, the CUR nanoparticles produced through either SAS or SEDS exhibit enhanced solubility and significantly higher dissolution rates compared to the crystalline CUR [40,41,42,43]. Furthermore, the micronization of CUR using the SEDS technique resulted in significant improvements in in vivo tests, indicating the potential for enhanced bioavailability and an improved therapeutic effect [44].

SCF technology using CO_2_ is also emerging as a promising approach for developing amorphous systems [45,46]. The supercritical carbon dioxide (scCO_2_) generated during the process facilitates the blending of the drug and polymer and promotes the mobility of amorphous polymers chains, thus allowing the process to be carried out at lower temperatures [47,48]. For achieving substances in the amorphous state using this method, it is vital that the amorphized substance is soluble in scCO_2_, and the choice of a polymer to which the amorphized substance exhibits affinity is crucial [48].

CUR displays solubility in scCO_2_ [49] and possesses lipophilic characteristics. As efficient carriers for enhancing the solubility of such compounds, hydrophilic polymers are acknowledged [30]. Among these polymers is poly(1-vinylpyrrolidone)-co-(vinyl acetate) (P(VP-co-VAc)) [50], which demonstrated successful utilization via the SCF method to achieve an amorphous system with another insoluble polyphenol, fisetin [46], and it was consequently selected for the study based on these considerations.

As per literature findings, to combine the advantages of ASDs with those of CAMs, ternary systems comprising a poorly soluble substance, a co-former, and a polymer can be formulated [51]. Therefore, to facilitate a comparison of how a ternary system would impact the physicochemical and biological properties of CUR in contrast to a binary system, a co-former was included.

As the co-former tryptophan (TRP) was selected, due to its well-known properties as an amino acid with advantageous co-formability, enabling the formation of co-amorphous systems with acidic, neutral, and basic drugs [52]. These systems exhibit good physical stability and improved dissolution profiles [53]. Additionally, TRP’s non-polar side chains are expected to facilitate its dissolution in scCO_2_. Moreover, considering CUR’s susceptibility to degradation in alkaline conditions [29,49], precautions were taken to prevent pH-related effects during solubility studies. TRP has previously demonstrated effectiveness as an amino acid in enhancing the solubility of ofloxacin in co-amorphous systems without impacting pH [54].

Finally, the combination of CUR with TRP holds potential benefits. TRP is an amino acid typically metabolized in the brain to 5-hydroxytryptamine (5-HTP) and serotonin (5-HT). However, in the presence of elevated levels of cytokines and glucocorticoids triggered by stress, TRP metabolism can shift towards the kynurenine pathway. This alternative pathway leads to the production of quinolinic acid, which exhibits neurotoxic effects on specific areas in the nervous system. It also leads to a reduction in serotonin production due to decreased availability of TRP for its normal synthesis. Indoleamine 2,3-dioxygenase (IDO) plays a pivotal role in the degradation of TRP to kynurenine metabolites. By inhibiting IDO, CUR may potentially prevent TRP depletion and the accumulation of neurotoxic kynurenine metabolites, which are associated with depressive symptoms. Furthermore, the anti-inflammatory and antioxidant properties of CUR may enhance its antidepressant effects.

Hence, the objectives of the study were twofold: firstly, to employ the SCF method for the synthesis and characterization of binary (CUR-(P(VP-co-VAc)) and ternary (CUR-TRP-(P(VP-co-VAc)) systems; secondly, to enhance the solubility of CUR and assess the impact of the properties of the binary and ternary systems on CUR, focusing on parameters such as dissolution rate, permeation across model artificial biological membranes (PAMPA), and antioxidant activity.

## 2. Results and Discussion

### 2.1. Systems Preparation

The initial phase of the research focused on acquiring amorphous systems containing CUR using the SCF method. The effectiveness of this method for achieving amorphization relies on the affinity between the amorphized substance and the polymer [48]. Therefore, to affirm the selection of the P(VP-co-VAc), which has been demonstrated in the literature to be effective in producing amorphous systems with poorly soluble polyphenol using the SCF method [46], we employed the melting-point depression method. This approach indicates that the melting point (Tm) of a crystalline substance decreases to a lower temperature when it is miscible with an amorphous polymer [45]. To determine the Tm of crystalline CUR within amorphous P(VP-co-VAc), a physical mixture was prepared at a 1:1 ratio, and differential scanning calorimetry (DSC) was utilized. The resulting diffractogram (Figure S1) is provided in Supplementary Materials. The measured Tm values were 180.4 °C for crystalline CUR and 165.4 °C for the physical mixture, indicating the potential miscibility of CUR with P(VP-co-VAc) due to the observed melting-point depression.

To determine the process temperature, we conducted a DSC study to ascertain the glass transition temperature (Tg) of P(VP-co-VAc), which was found to be 118.5 °C. Considering the plasticizing effect of scCO_2_ on the polymer, resembling the impact of heat, which decreases Tg and enhances the mobility of polymer chains [55], the process temperature was established at 80 °C.

Another consideration, which influenced the selection of method parameters, is the solubility in scCO_2_. The solubility of CUR in scCO_2_ is complex and influenced by temperature and pressure. Additionally, the effect of temperature and pressure on CO_2_ as a solvent must be considered. Generally, higher temperatures and pressures are required for improved solubility, as reduced fluid density at higher temperatures and lower pressures leads to decreased solubility [49]. Therefore, the process was conducted at 6000 psi to achieve optimal solubility in scCO_2_.

To identify an optimal system in terms of complete amorphousness and the enhancement of CUR solubility, systems with varying CUR loadings were prepared. The amorphous state of the obtained samples was confirmed using X-ray powder diffraction (XRPD). For binary systems containing 10%, 15%, and 20% CUR, crystalline peaks were absent in the diffractograms at 10% and 15% CUR content (Figure S2, Supplementary Materials). These systems were subjected to high-performance liquid chromatography (HPLC) analysis to assess solubility improvement. A solubility enhancement of 9.87% ± 0.85% was observed for the system containing 10% CUR compared to the system with 15% CUR.

Consequently, a ternary system with TRP was chosen for the system with 10% CUR content. Systems containing 5%, 10%, and 15% TRP addition were prepared. A sample in the amorphous state was obtained for systems with 5% and 10% TRP addition (Figure S3, Supplementary Materials). These systems underwent HPLC analysis to determine their effect on improving CUR solubility. A 13.01% ± 0.95% higher solubility of CUR was observed in the system with 5% TRP addition compared to the system with 10% TRP addition. Consequently, two systems were selected for further study: one comprising 10% CUR and P(VP-co-VAc) (binary CUR-P(VP-co-VAc) amorphous system) and one comprising 10% CUR, 5% TRP, and P(VP-co-VAc) (ternary CUR-TRP-P(VP-co-VAc) amorphous system).

### 2.2. X-Ray Powder Diffraction

**Figure 1:**
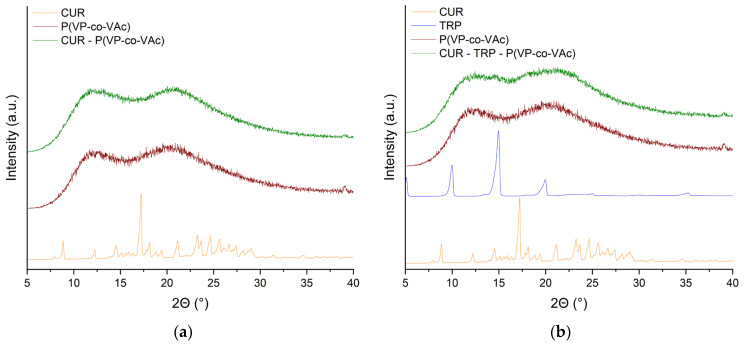
XRPD diffraction patterns of CUR, P(VP-co-VAc), and binary CUR-P(VP-co-VAc) amorphous system (**a**), CUR, TRP, P(VP-co-VAc), and ternary CUR-TRP-P(VP-co-VAc) amorphous system (**b**).

XRPD was used to confirm the absence of characteristic diffraction peaks associated with crystalline structures, thereby indicating the presence of an amorphous phase. The XRPD diffractograms of the polymer P(VP-co-VAc) clearly indicated its amorphous nature, as expected. Conversely, the diffractograms of crystalline CUR and TRP exhibited distinct and characteristic crystalline peaks, confirming their crystalline structures. These peaks, albeit with reduced intensity due to the presence of the amorphous polymer, were also observed in physical mixtures, both binary and ternary systems (see Figure S4a,b) as well as in the prepared CUR-TRP system (Figure S5, Supplementary Materials). This observation indicated that obtaining a co-amorphous CUR-TRP system solely through the use of TRP as a co-former via the SCF method was not feasible, necessitating the addition of a polymer to preserve the amorphous state. The complete disappearance of crystalline peaks, indicating full amorphization, was observed for the binary CUR-P(VP-co-VAc) and ternary CUR-TRP-P(VP-co-VAc) amorphous systems (Figure 1).

### 2.3. Differential Scanning Calorimetry

**Figure 2:**
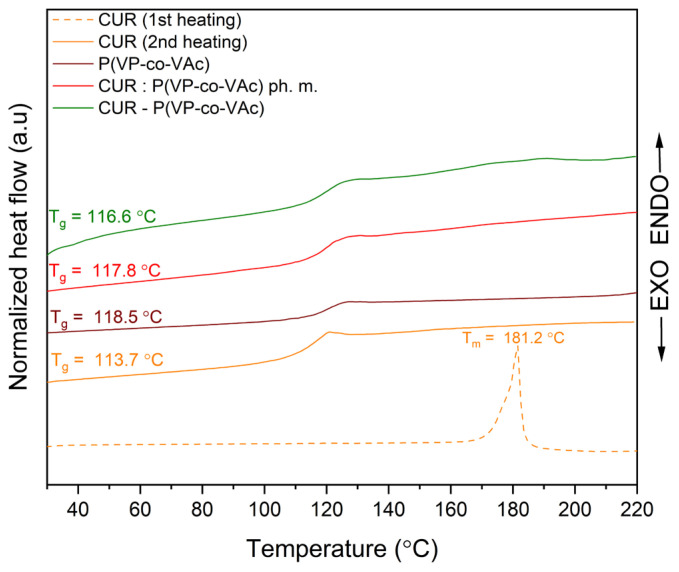
DSC thermograms of CUR, P(VP-co-VAc), binary CUR:P(VP-co-VAc) physical mixture, and binary CUR-P(VP-co-VAc) amorphous system.

**Figure 3:**
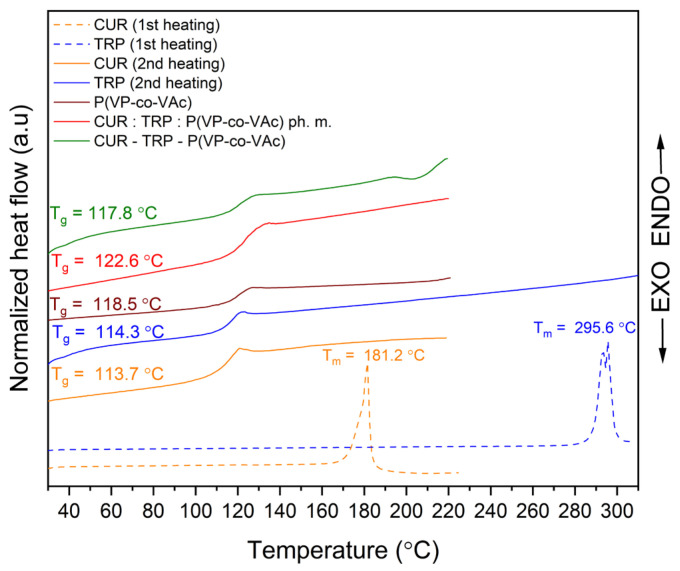
DSC thermograms of CUR, TRP, P(VP-co-VAc), ternary CUR:TRP:P(VP-co-VAc) physical mixture, and ternary CUR-TRP-P(VP-co-VAc) amorphous system.

DSC, in conjunction with XRPD, is utilized to investigate the amorphous state of crystalline substances. As a substance undergoes amorphization, its Tm disappears from the thermogram. The resulting thermograms of CUR, TRP, P(VP-co-VAc), physical mixtures, and the obtained systems are depicted in Figure 2 and Figure 3.

The crystalline CUR exhibits a sharp endothermic peak in the DSC curve at approximately 181.2 °C, representing its Tm, consistent with data reported in the literature [56]. The Tm of crystalline TRP was observed at 295.5 °C. In both the binary and ternary systems, where the amorphous state was previously validated through XRPD analysis, a single glass transition was noted (at 116.6 °C for the binary CUR-P(VP-co-VAc) amorphous system and at 117.8 °C for the ternary CUR-TRP-P(VP-co-VAc) amorphous system), along with the absence of Tm peaks for both CUR and TRP. However, while the XRPD analysis indicated the amorphous nature of these systems, this observation cannot be conclusively supported on this basis alone, as similar effects were observed for both binary and ternary physical mixtures. This is attributed to the dominant presence of the amorphous polymer, which, in a higher proportion compared to other components, overshadows the interaction effects [45].

### 2.4. Fourier-Transform Infrared Spectroscopy

**Figure 4:**
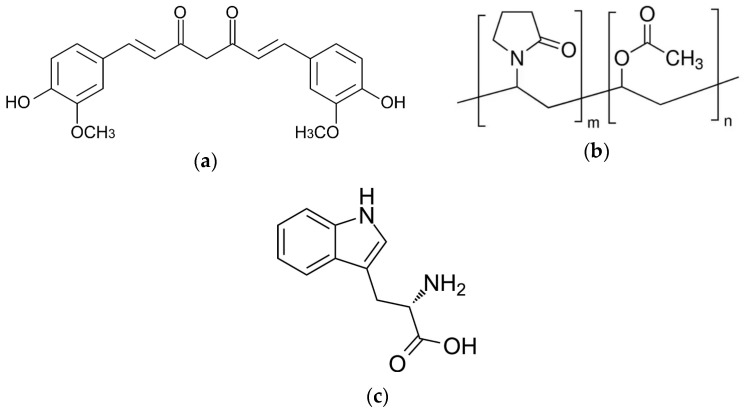
Structure of (**a**) CUR, (**b**) P(VP-co-VAc), and (**c**) TRP.

FT-IR analysis is often used to indicate the interactions responsible for the development of amorphous systems [57,58,59,60]. For this reason, FT-IR-ATR analysis was used in the MIR region (400–4000 cm^−1^) to identify the interactions responsible for maintaining the amorphous state of CUR in binary and ternary systems. The chemical structure of CUR, P(VP-co-VAc), and TRP are shown in Figure 4.

**Figure 5:**
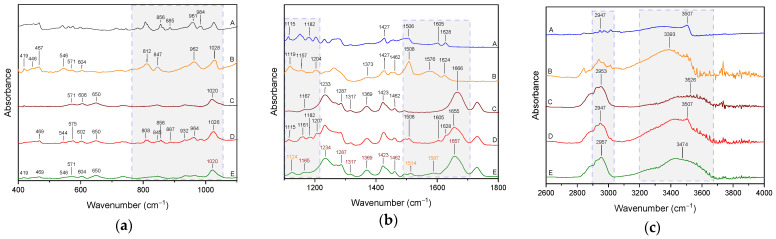
FT-IR analysis: CUR (A), amorphous CUR (B), P(VP-co-VAc) (C), binary CUR:P(VP-co-VAc) physical mixture (D), binary CUR-P(VP-co-VAc) amorphous system (E); (**a**) range 400–1100 cm^−1^, (**b**) range 1100–1800 cm^−1^, (**c**) range 2600–4000 cm^−1^.

In the range of 400–1100 cm^−1^ (Figure 5a) for the binary CUR:P(VP-co-VAc) physical mixture, we observe bands originating from CUR and P(VP-co-VAc).

In the case of a binary CUR-P(VP-co-VAc) amorphous system, we observe the disappearance of bands in the range of ~800–1000 cm^−1^ corresponding to CUR. Moreover, the FT-IR spectrum is dominated by bands corresponding to P(VP-co-VAc), with the most characteristic band at 1020 cm^−1^ corresponding to the vibrations of C-N bonds. Nevertheless, two bands that can be assigned to amorphous CUR are also present (419 cm^−1^ and 469 cm^−1^). No shifts of these bands are observed [57,61].

Similarly, in the range of 1100–1800 cm^−1^ (Figure 5b), in the binary CUR:P(VP-co-VAc) physical mixture, we observe the sum of the bands of the individual components, whereas in the binary CUR-P(VP-co-VAc) amorphous system, the bands characteristic of P(VP-co-VAc) predominate. Nevertheless, changes are observed in the nature of the spectrum of the binary CUR-P(VP-co-VAc) amorphous system, which indicate interactions between CUR and P(VP-co-VAc). The bands at 1666 cm^−1^ (C=O stretching, N-vinylpyrrolidone), 1233 cm^−1^ (lactone structure) and 1167 cm^−1^ observed in the spectrum of P(VP-co-VAc) are shifted to 1657 cm^−1^, 1234 cm^−1^, and 1165 cm^−1^ in the spectrum of the binary CUR-P(VP-co-VAc) amorphous system. Bands characteristic for amorphous CUR at 1119 cm^−1^ (C-C stretching), 1508 cm^−1^ (C=O stretching and bending of C=O and C=C), and 1576 cm^−1^ (C=C stretching + C-C stretching + C-O-H bending) are shifted to 1124 cm^−1^, 1514 cm^−1^, and 1587 cm^−1^ [57,61,62].

In the range of 2600–4000 cm^−1^ (Figure 5c), the spectrum of the binary CUR-P(VP-co-VAc) amorphous system consists exclusively of bands originating from P(VP-co-VAc). Shifts of two characteristic bands are observed (at 2957 cm^−1^ corresponding to asymmetric CH_2_ stretching and at 3474 cm^−1^ corresponding to O-H and NH stretching) [57,61].

The disappearance of the characteristic CUR bands, the change in the shape and/or shifts of the CUR and/or P(VP-co-VAc) bands indicate the formation of an interaction between CUR and P(VP-co-VAc). P(VP-co-VAc) possesses two hydrogen bond acceptor groups: at 1666 cm^−1^, there is C=O stretching of N-vinylpyrrolidone, and at 1732 cm^−1^, there is a C=O group of the vinyl acetate [57]. The shift of the band observed in the CUR spectrum at 1576 cm^−1^ (C-O-H bending) and the P(VP-co-VAc) band observed at 1666 cm^−1^ (C=O stretching of N-vinylpyrrolidone) confirms the formation of hydrogen bonds between the C-O-H group of CUR and C=O P(VP-co-VAc).

**Figure 6:**
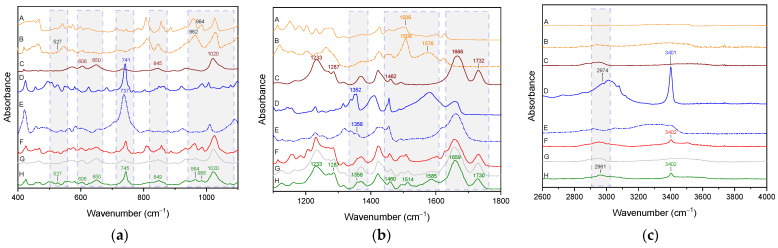
FT-IR analysis: CUR (A), amorphous CUR (B), P(VP-co-VAc) (C), TRP (D), amorphous TRP (E), ternary CUR:TRP:P(VP-co-VAc) physical mixture (F), binary CUR-P(VP-co-VAc) amorphous system (G), ternary CUR-TRP-P(VP-co-VAc) amorphous system (H); (**a**) range 400–1100 cm^−1^, (**b**) range 1100–1800 cm^−1^, (**c**) range 2600–4000 cm^−1^.

The spectrum of the ternary CUR:TRP:P(VP-co-VAc) physical mixture in the 400–4000 cm^−1^ range is a composite of bands corresponding to CUR, P(VP-co-VAc) and TRP (see Figure 6).

The spectrum of the ternary CUR-TRP-P(VP-co-VAc) amorphous system, analogously to the binary CUR-P(VP-co-VAc) amorphous system, is dominated by characteristic P(VP-co-VAc) bands. In the 400–1100 cm^−1^ range (Figure 6a), one can distinguish, in addition to the P(VP-co-VAc) bands, bands corresponding to amorphous CUR (deformation of CUR molecule: at 606 cm^−1^, 650 cm^−1^, 964 cm^−1^, and C-H wagging at 849 cm^−1^), CUR or TRP (deformation of CUR molecule or C-N stretching at 986 cm^−1^) and amorphous TRP (C-H out-of-plane angle bending vibrations (ring) at 745 cm^−1^). A shift of P(VP-co-VAc) bands (from 845 cm^−1^ to 849 cm^−1^), amorphous CUR (deformation of CUR molecule from 962 cm^−1^ to 964 cm^−1^), CUR or TRP (deformation of CUR molecule or C-N stretching in TRP, from 984 cm^−1^ to 986 cm^−1^), and amorphous TRP (C-H out-of plane angle bending vibrations (ring), from 737 cm^−1^ to 745 cm^−1^) is observed. In the 1100–1800 cm^−1^ range (Figure 6b), P(VP-co-VAc) band shifts (O-H shear from 1462 cm^−1^ to 1460 cm^−1^, C=O stretching (N-vinylpyrrolidone) from 1666 cm^−1^ to 1649 cm^−1^, C=O stretching (vinyl acetate) from 1732 cm^−1^ to 1730 cm^−1^ and amorphous CUR (C-O stretching and C-C stretching from 1508 cm^−1^ to 1514 cm^−1^, C=C stretching, C-C stretching, and C-O-H bending from 1576 cm^−1^ to 1585 cm^−1^) are observed. In the range of 2600–4000 cm^−1^ (Figure 6c), the changes in the nature of the spectrum of the ternary CUR-TRP-P(VP-co-VAc) amorphous system involve only the band corresponding to the stretching vibrations of the C-H TRP group, which is visible in the system with a maximum at 2961 cm^−1^ [57,61,62,63].

Band shifts observed in the CUR spectrum at 1576 cm^−1^ (C-O-H bending) and 1508 cm^−1^ (C=O stretching and bending of C=O and C=C), in the P(VP-co-VAc) spectrum at 1666 cm^−1^ (C=O stretching of N-vinylpyrrolidone) and 1732 cm^−1^ (C=O group of the vinyl acetate), and in the TRP spectrum at 737 cm^−1^ (C-H out-of-plane angle bending vibrations (ring)) and 2961 cm^−1^ (C-H stretching) confirm the formation of hydrogen bonds (i) between the C-O-H group of CUR and the C=O group of P(VP-co-VAc), (ii) between the C-H TRP group and C-O of CUR, and/or (iii) between the C=O P(VP-co-VAc) group and C-H TRP. Because P(VP-co-VAc) has two hydrogen bond acceptor groups, it can form a hydrogen bond with CUR via the C=O group of N-vinylpyrrolidone (confirmed for the binary CUR-P(VP-co-VAc) amorphous system) and with TRP via the C=O group of vinyl acetate.

### 2.5. Scanning Electron Microscopy

**Figure 7:**
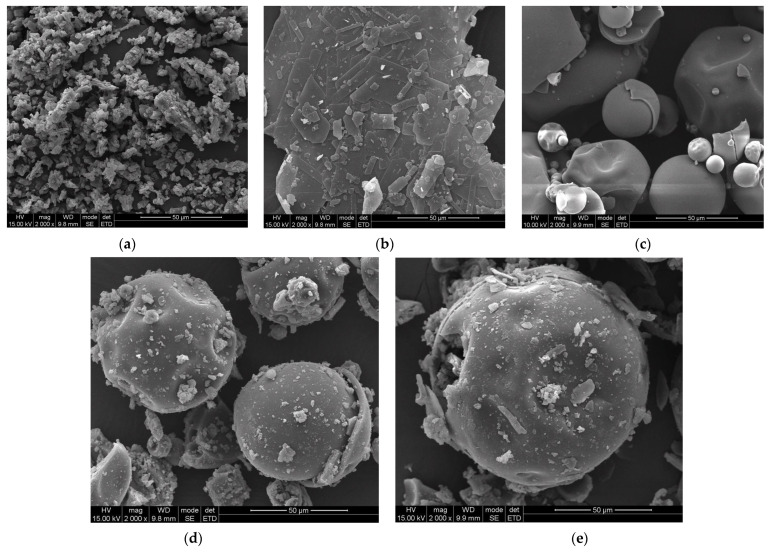

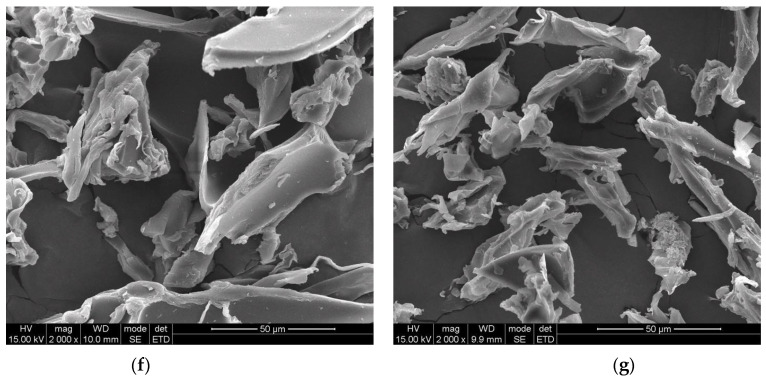
SEM micrographs of CUR (**a**), TRP (**b**), P(VP-co-VAc) (**c**), binary CUR:P(VP-co-VAc) physical mixture (**d**), ternary CUR:TRP:P(VP-co-VAc) physical mixture (**e**), binary CUR-P(VP-co-VAc) amorphous system (**f**), and ternary CUR-TRP-P(VP-co-VAc) amorphous system (**g**).

The morphology of powder particles from raw materials, physical mixtures, and the resulting amorphous systems is illustrated in Figure 7. SEM observations reveal distinct differences in the shape and size of the initial powders, their physical mixtures, and the amorphized materials. CUR particles (Figure 7a) exhibit an irregular shape with sizes ranging widely—from a few microns to approximately 40 µm. TRP particles (Figure 7b) have a flaky shape, with most particles ranging in size from 40 to 60 µm. P(VP-co-VAc) particles (Figure 7c) are predominantly globular in shape, along with fractured particles likely caused during the analysis, with sizes varying between 10 and 60 µm.

Both physical mixtures—binary CUR:P(VP-co-VAc) (Figure 7d) and ternary CUR:TRP:P(VP-co-VAc) (Figure 7e)—maintain the same morphology as the initial powders. Smaller CUR and TRP particles adhere to the larger P(VP-co-VAc) particles. The amorphization process, however, alters the morphology of the powder particles. Both amorphous systems, shown in Figure 7f,g, exhibit similar morphologies distinct from the initial materials. Their shapes are irregular, and the particle size of the binary CUR-P(VP-co-VAc) amorphous system is larger than that of the ternary CUR-TRP-P(VP-co-VAc) amorphous system.

### 2.6. Apparent Solubility Studies

Various studies have reported the solubility of CUR in water, ranging from as low as 0.6 µg·mL^−1^ [64] and 0.25 µg·mL^−1^ [65] to 0.011 µg·mL^−1^ [65]. The estimated value is 3.21 µg·mL^−1^ at 25 °C [66]. Consequently, CUR can be categorized as practically insoluble [67]. This assertion is further supported by our study, wherein the solubility of crystalline CUR in water was found to be 3.17 µg·mL^−1^.

**Figure 8:**
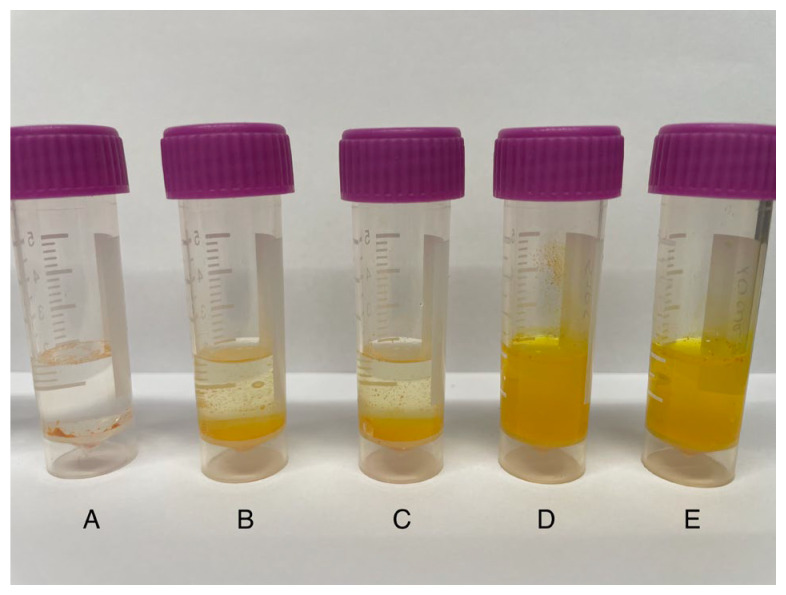
Photographs of (**A**) CUR, (**B**) binary CUR:P(VP-co-VAc) physical mixture, (**C**) ternary CUR:TRP:P(VP-co-VAc) physical mixture, (**D**) binary CUR-P(VP-co-VAc) amorphous system, (**E**) ternary CUR-TRP-P(VP-co-VAc) amorphous system during solubility study at 180 min in water.

The dissolution process, which could be visually observed during the solubility study, is depicted in Figure 8.

It was found that both the raw CUR and the physical mixtures settled at the bottom in the form of aggregates (Figure 8A–C). Conversely, binary CUR-P(VP-co-VAc) and ternary CUR-TRP-P(VP-co-VAc) amorphous systems produced using the SFC method, which were confirmed amorphous by XRPD, dispersed easily and did not form clusters in the medium (Figure 8D,E). Amorphous forms typically have higher solubility than crystalline forms due to structural differences. The lack of defined structure in amorphous forms leads to higher surface areas, lower energy requirements for disruption, and easier penetration by solvent molecules, all contributing to their increased solubility compared to crystalline forms.

These observations were reflected and confirmed in the study using the HPLC method. The solubility of CUR did not show improvement when in crystalline form within physical mixtures; only the transformation into an amorphous form within the resultant systems enhanced CUR solubility. In both the binary CUR-P(VP-co-VAc) and ternary CUR-TRP-P(VP-co-VAc) amorphous systems, owing to the improvement in CUR solubility and in consideration of the solubility values acquired, CUR can be classified as very slightly soluble. The enhancement in solubility was significantly more pronounced within the ternary system, showing a statistically significant difference (*p* < 0.05) (Table 1).

The solubility of CUR is influenced by pH. While CUR demonstrates better solubility under alkaline conditions, its stability is compromised at high pH [68,69]. In the ternary CUR-TRP-P(VP-co-VAc) system created with the use of a co-former, efforts were made to counteract the impact of the co-former on the pH of the solutions obtained in the solubility study. The choice of the amino acid is crucial in this context, as basic amino acids like lysine or arginine, despite their favorable co-formulation properties, are known to raise solution pH levels [54,70]. In situations where pH changes are insignificant or unwanted, selecting an amino acid that has no noticeable effect on pH becomes essential. TRP was found to have no such pH-altering effect [54]. To confirm this observation and eliminate potential pH-related influences on system solubility, pH measurements were carried out on solutions obtained from the solubility test. The collected data are presented in Table 2.

Due to the lack of changes in pH levels, the potential impact of pH variations as a contributing factor to the observed increase in solubility was ruled out. Instead, the improvement in solubility was primarily attributed to the transformation of CUR into an amorphous state, as confirmed in XRPD study. The lack of a defined crystal lattice in amorphous forms reduces the energy barriers to dissolution, making them more readily soluble in aqueous environments [71]. Furthermore, since many materials produced using supercritical fluids exhibit particle-size distributions in the nanometer range [40,42,72], it cannot be excluded that the observed increase in the solubility of processed CUR was also influenced by its reduced particle size and increased surface area.

Moreover, the enhancement of solubility observed in the ternary CUR-TRP-P(VP-co-VAc) amorphous system in comparison to the binary CUR-P(VP-co-VAc) amorphous system was found to be statistically significant. Within this system, the interactions between CUR and TRP were validated at the molecular level. This suggests that the creation of a co-amorphous system dispersed within the polymer could have provided an additional advantageous effect in improving the solubility of CUR. Moreover, the presence of TRP, a highly soluble water amino acid [73], may have further influenced this enhancement. The existing literature has indicated that as the solubility of the co-former utilized increases, there is a corresponding rise in the solubility of the co-amorphous system [70,74]. Furthermore, as demonstrated by the SEM analysis, the particle size of the ternary CUR-TRP-P(VP-co-VAc) amorphous system is smaller than that of the binary CUR-P(VP-co-VAc) amorphous system. The amorphous nature and smaller particle size may contribute to higher apparent solubility.

### 2.7. Dissolution-Rate Studies

**Figure 9:**
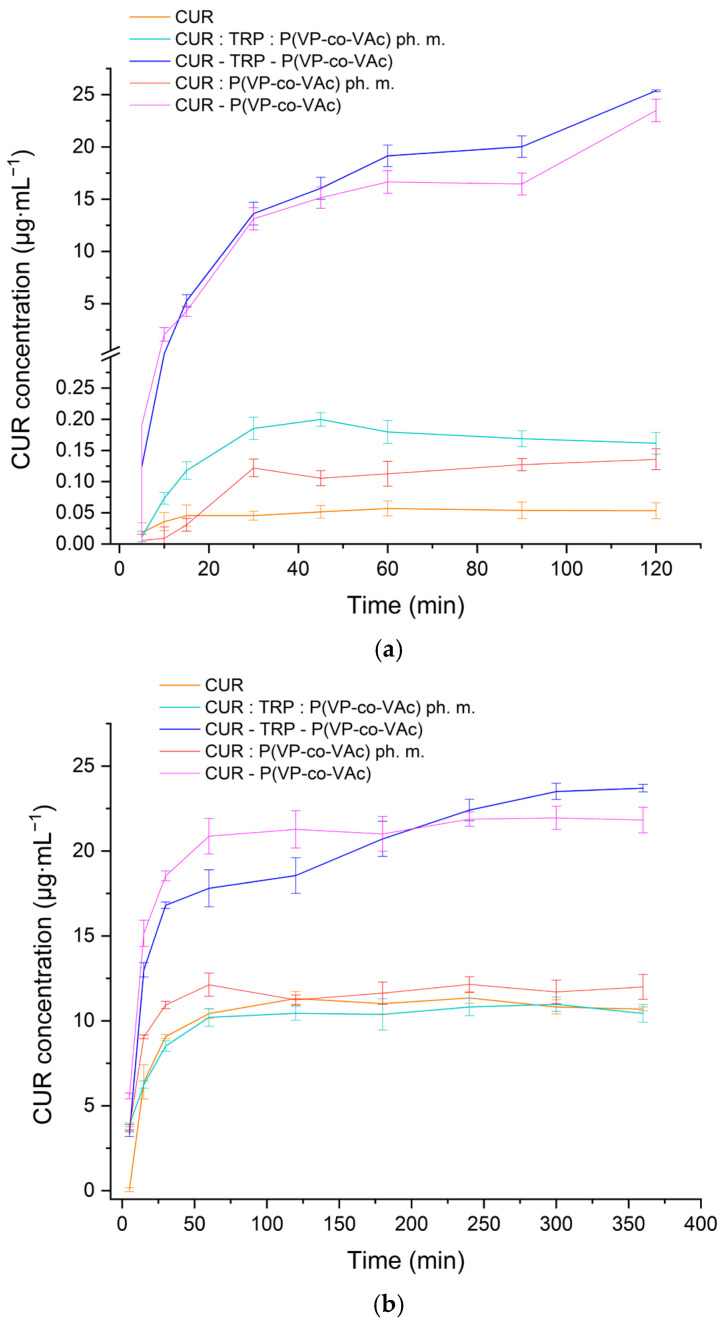
Dissolution profiles of CUR, physical mixtures, and obtained amorphous systems in a pH of 1.2 (**a**) and in a pH of 6.8 (**b**).

The dissolution rate of the crystalline CUR, physical mixtures and obtained amorphous systems were determined in the media at pH 1.2 and 6.8. The outcomes depicted as dissolution-rate profiles are illustrated in the Figure 9.

In both types of media, the crystalline form of CUR demonstrated a notably lower dissolution rate when compared to its amorphous state within the studied systems. Additionally, the influence of pH on the dissolution process was clearly visible. CUR is recognized for its limited solubility in water, particularly evident at lower pH levels due to alterations in its molecular configuration under acidic conditions [68].

The dissolution profiles are consistent with those described in the literature for relatively low drug loads in systems based on the hydrophilic polymer P(VP-co-VAc) [75,76]. Similar behavior was also reported in studies on ASDs utilizing β-lactoglobulin as the carrier [77]. 

An essential deduction from the dissolution profiles pertains to whether the systems were able to achieve and uphold supersaturation. This issue becomes particularly critical in co-amorphous systems, where the maintenance of supersaturation seems to depend on stabilization mechanisms distinct from those in systems where polymers stabilize the amorphous substance [34].

The preservation of supersaturation in co-amorphous systems involving amino acids is intricate, with suggestions that the interaction between the amorphized substance and the co-former plays a significant role, impeding recrystallization in the presence of water that acts as a plasticizer. The transformation back to a more stable crystalline state from supersaturated drug solutions can also encourage recrystallization, a recognized challenge during release studies [70,78]. Even a small addition of polymer can have a beneficial impact on preserving the supersaturation state [79].

The utilization of FT-IR analysis confirmed the presence of interactions between CUR and TRP in the ternary CUR-TRP-P(VP-co-VAc) amorphous system obtained. Nevertheless, the inability to generate a co-amorphous system of CUR with TRP using the SCF method without the incorporation of polymer hindered the evaluation of its capability to uphold supersaturation during dissolution studies. There was no significant distinction observed between binary CUR-P(VP-co-VAc) and ternary CUR-TRP-P(VP-co-VAc) amorphous systems in terms of their ability to maintain supersaturation, suggesting that the polymer itself is likely the primary factor contributing to this effect. Nonetheless, the introduction of TRP in the ternary CUR-TRP-P(VP-co-VAc) amorphous system led to a slight enhancement in apparent solubility after 60 min in HCl and after 250 min in buffer compared to the binary CUR-P(VP-co-VAc) amorphous system, aligning with findings from previous solubility studies.

In the case of HCl, the amorphous systems remained supersaturated for approximately two hours, corresponding to the usual retention time in the stomach. However, it is apparent that this two-hour duration was insufficient to reach a plateau. In pH 6.8 buffer, both systems sustained supersaturation for around 6 h, which aligns with the typical duration of intestinal content residence in the duodenum.

### 2.8. In Vitro Parallel Artificial Membrane-Permeability Assay

**Figure 10:**
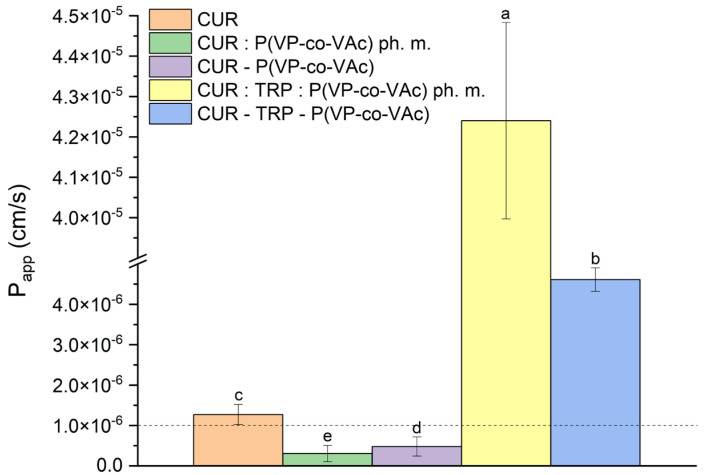
The results of PAMPA GIT assay. The statistically significant values are indicated on the figure by letters from ‘a’–‘e’, where ‘a’ represents the highest value (*p* < 0.05).

Following oral intake, the absorption process predominantly depends on the substance’s solubility, dissolution rate, and gastrointestinal permeability. To evaluate the impact of enhanced solubility and dissolution rate in the developed amorphous systems on the passive diffusion of CUR, the parallel artificial membrane-permeability assay (PAMPA) was employed. This method is used to predict transcellular drug absorption by measuring the flux of compounds between two aqueous compartments separated by a microporous filter infused with lipids. The results are presented in Figure 10.

The logP value of CUR is reported as ~3.0 [80]. It is therefore expected that its lipophilicity will support effective penetration of cell membranes. The permeability coefficient (*Papp*) value for raw CUR obtained in our study was 1.27 × 10^−6^ ± 2.48 × 10^−7^ cm·s^−1^. Therefore, it can be classified as a highly permeable compound, which is consistent with the literature data [14,33]. The *Papp* value of CUR in the binary CUR:P(VP-co-VAc) physical mixture was 3.06 × 10^−7^ ± 2.02 × 10^−7^ cm·s^−1^, while in the binary CUR-P(VP-co-VAc) amorphous system, it was 4.79 × 10^−7^ ± 2.37 × 10^−7^ cm·s^−1^. In both scenarios, the permeability of CUR decreased, indicating its classification as a medium-permeable compound. In the binary CUR:P(VP-co-VAc) physical mixture, where CUR exhibited the lowest *Papp* value, the polymer did not demonstrate permeability-enhancing properties. In the case of the binary CUR-P(VP-co-VAc) amorphous system, an enhancement in CUR solubility was noted alongside a decline in permeability. Similar instances have been documented in the existing literature. Factors like increased solubility leading to the decreased hydrophilicity of the compound, molecular interactions with cellular membranes, or higher levels of ionized substances in the solution are suggested as potential factors contributing to the decrease in membrane-permeation characteristics in this basic membrane-permeability model [70,81,82].

Interestingly, this trend was not observed in the ternary CUR-TRP-P(VP-co-VAc) amorphous system. Here, despite the improved solubility, the high permeability of CUR was maintained. The *Papp* value for CUR in this system was 4.61 × 10^−6^ ± 2.93 × 10^−7^ cm·s^−1^, slightly superior to that of pure CUR. The presence of TRP in this system may have played a role in maintaining this high permeability by potentially acting as a permeability enhancer. The mechanism behind this remains elucidated; however, amino acids have the potential to serve as facilitators in enhancing the permeability of other substances through membranes. This has been observed for amino acids like TRP, arginine and cysteine [70,82,83]. The impact of TRP as a permeability enhancer was also observed in the ternary CUR:TRP:P(VP-co-VAc) physical mixture, where the *Papp* value was 4.24 × 10^−5^ ± 2.43 × 10^−6^ cm·s^−1^.

### 2.9. Biological Activity Assessment

In order to evaluate the impact of enhanced solubility of CUR in the developed amorphous systems on its biological properties, tests were carried out to assess its activity concerning its capacity to neutralize DPPH radical and its capability to inhibit the activity of butyrylcholinesterase (BChE). The results are presented in Table 3.

CUR has been studied for its free radical scavenging activity, which is a key aspect of its antioxidant properties. It contains phenolic hydroxyl groups that can donate hydrogen atoms to free radicals, neutralizing their reactivity [84]. However, its low solubility can limit its effectiveness. In our study, the efficacy of crystalline CUR in reducing DPPH radical was 13.38 ± 0.42%. No enhancement was observed in the physical mixtures prepared, with reductions of 8.31 ± 0.58 and 9.79 ± 0.89% noted for the binary CUR:P(VP-co-VAc) and ternary CUR:TRP:P(VP-co-VAc) physical mixtures, respectively. A notable improvement was observed in the amorphous systems. However, neither of them can be deemed superior; both demonstrated around 88% scavenging inhibition of the radicals at concentrations achieved in the solubility studies.

CUR is also known for its neuroprotective potential associated with its ability to inhibit cholinesterases [85]; however, its utilization in this case is also hindered by low solubility [86]. When dissolved in water, CUR exhibited a 2.97 ± 0.87% inhibition of BChE. Physical mixtures did not enhance enzyme inhibition. A significant enhancement in inhibiting BChE was observed in the amorphous systems. In the binary CUR-P(VP-co-VAc) amorphous system, CUR demonstrated a 46.64 ± 0.74% suppression of BChE activity, while in the ternary CUR-TRP-P(VP-co-VAc) amorphous system, this suppression reached 74.54 ± 1.88%. The inhibitory activity was attributed to CUR, as TRP, a water-soluble compound present in the ternary CUR-TRP-P(VP-co-VAc) amorphous system, did not exhibit BChE inhibitory properties.

## 3. Materials and Methods

### 3.1. Materials

Curcumin (purity > 95%) was supplied by Xi’an Tian Guangyuan Biotech Co. (Shenyang, China). Tryptophan was sourced from TCI Chemicals (Portland, OR, USA). Sigma-Aldrich (St. Louis, MO, USA) provided poly(1-vinylpyrrolidone)-co-(vinyl acetate), 2,2-diphenyl-1-picrylhydrazyl, and 5,5-dithio-bis-(2-nitrobenzoic acid). HPLC-grade acetonitrile came from J. T. Baker (Center Valley, PA, USA). Water of ultra-high purity was prepared using a Direct-Q 3 UV Merck Millipore purification system (Burlington, MA, USA). Chempur (Piekary Slaskie, Poland) provided the analytical-grade HCl 1 N. Potassium dihydrogen phosphate, sodium hydroxide, sodium chloride, acetic acid, and dimethyl sulfoxide were obtained from Avantor Performance Materials Poland S.A. (Gliwice, Poland). All reagents for the PAMPA assay, including GIT lipid solution, Prisma HT, and Acceptor Sink Buffer, were acquired from pION (Forest Row, East Sussex, UK). Methanol was sourced from POCH (Gliwice, Poland). Magnesium chloride hexahydrate, Trizma^®^ hydrochloride, Trizma^®^ base, butyrylcholinesterase from equine serum, and butyrylcholine iodide were delivered from Sigma-Aldrich (Schnelldorf, Germany).

### 3.2. Systems Preparation

The preparation of the systems was based on the method developed by Sip et al. [46] with minor modifications. Briefly, the physical mixtures were ground in a mortar for 5 min with 3 g and then transferred to a steel vessel and placed in the SFT-120 apparatus (Supercritical Fluid Technologies, Inc., Newark, DE, USA). Samples were subjected to a process at 80 °C and 6000 psi pressure for 2 h. Following the completion of the experiment, carbon dioxide was discharged from the apparatus; then, the sample was removed from the vessel and homogenized in a Tube Mill 100 Control (IKA, Warsaw, Poland) for 1 min at 5000 rpm.

### 3.3. X-Ray Powder Diffraction

Using X-ray powder diffraction on a Bruker AXS D2 Phaser diffractometer (Bruker, Germany) with a CuKα X-ray radiation source (30 kV, 10 mA, λ = 1.54060 Å), the crystalline structure of all samples was examined. Under ambient conditions, data were gathered at a step size of 0.02° and a counting rate of 2 s per step throughout a range of 2θ diffraction angles, from 5° to 40°. Origin 2021b (OriginLab Corporation, Northampton, MA, USA) software was used to process and illustrate the data.

### 3.4. Differential Scanning Calorimetry

A DSC 214 Polyma differential scanning calorimeter (Netzsch, Selb, Germany) was utilized to carry out a differential scanning calorimetry analysis. The reference was a blank aluminum DSC pan, while 9–10 mg of the samples was placed in the sealed pans with a lid hole.

With minor modifications, thermal runs for the melting-point depression method were carried out in accordance with Guo et al. [45], specifically over a temperature range of 30–230 °C at a heating rate of 5 °C·min^−1^.

The melting point and glass transition temperature of physical mixtures and obtained systems were determined through a sequence of temperature changes. Initially, the samples were subjected to a temperature range from 30 °C to 150 °C with a heating rate of 40 °C·min^−1^ and held isothermally for 5 min, in order to remove water. In the subsequent step, the temperature decreased to 30 °C at a cooling rate of 40 °C·min^−1^. Next, the temperature was raised to 230 °C at a heating rate of 10 °C·min^−1^. The samples were then cooled again to 5 °C at a cooling rate of 40 °C·min^−1^ and finally heated to 230 °C with a heating rate of 40 °C·min^−1^.

The thermal runs conducted for CUR were as follows: heating from 30 °C to 230 °C with a heating rate of 10 °C·min^−1^, cooling to 5 °C with a cooling rate of 40 °C·min^−1^ and heating back to 230 °C with a heating rate of 40 °C·min^−1^.

A sample of TRP was initially heated from 30 °C to 315 °C with a heating rate of 10 °C·min^−1^ and held isothermally for 5 min. The Tg was determined by cooling the melt to 30 °C with a cooling rate of 40 °C·min^−1^ and then heating it back to 315 °C with a heating rate of 40 °C·min^−1^.

The polymer sample required a first step of removing water, involving heating from 25 °C to 100 °C with a heating rate of 10 °C·min^−1^ and holding isothermally for 10 min. In the subsequent step, the temperature increased to 200 °C at a heating rate of 40 °C·min^−1^. Following that, the temperature was decreased to −10 °C with a cooling rate of 40 °C·min^−1^ and finally heated to 260 °C with a heating rate of 40 °C·min^−1^.

All analyses were carried out under a nitrogen atmosphere with a flow rate of 250 mL·min^−1^.

Proteus 8.0 (Netzsch, Selb, Germany) was used to analyze the acquired data, and Origin 2021b (OriginLab Corporation, Northampton, MA, USA) was used for presenting the results.

### 3.5. Fourier-Transform Infrared Spectroscopy

Mid-infrared spectra were measured at a resolution of 4 cm^−1^ (400 scans) using an IRTracer-100 spectrophotometer equipped with a diamond ATR module. The wave number range of the spectrum was 4000–400 cm^−1^. The spectra were acquired and processed using the LabSolution IR version 1.86 SP2 software (Warsaw, Poland).

### 3.6. Scanning Electron Microscopy

Microscopic observations of the initial and amorphized powders were carried out using an Inspect S scanning electron microscope (FEI, Eindhoven, The Netherlands) equipped with an Everhardt-Thornley detector. The observations were conducted at an accelerating voltage of 15 kV.

### 3.7. Chromatographic Conditions

The previously published method [14] was followed with minor modifications to determine the CUR content using HPLC. In compliance with ICH guidelines, the procedure was fully validated. Figure S6 and Table S1 (Supplementary Materials) contain the chromatogram of CUR and validation parameters for the method developed. A diode array detector-equipped Shimadzu Nexera system (Shimadzu Corp., Kyoto, Japan) was used. Samples were introduced into the 5 μm Dr Maisch ReproSil-Pur Basic-C18 column (Ammerbuch-Entringen, Germany) of 250 mm × 4.6 mm size. The study-specific injection volume was determined to be 1 μL for the apparent solubility test, 10 μL for permeability test, and 20 μL for the powder dissolution study. The column was fixed at a temperature of 30 °C. The mobile phase was a degassed blend of acetonitrile and 1% acetic acid (55:45, *v*/*v*). At 1 mL·min^−1^, the flow was isocratic. The wavelength of the analysis was 425 nm.

### 3.8. Apparent Solubility Studies

Solubility studies were conducted using distilled water. A quantity of samples in excess of its expected saturated solubility were administered along with 2 mL of a medium. The samples were then put in a laboratory incubator (MaxQ 4450; Thermo Scientific, Waltham, MA, USA) with the agitation speed set to 75 rpm, maintained at 25 °C, and shielded from light. Following a three-hour period, the solutions were filtered through a 0.22 μm PTFE syringe filter before being introduced into the HPLC to analyze the CUR content.

### 3.9. Dissolution-Rate Studies

Dissolution-rate studies were performed using a paddle-equipped apparatus (Agilent, Santa Clara, CA, USA). Samples, including 60 mg of pure CUR or physical mixtures or systems in an amount equivalent to 60 mg of CUR, were placed in gelatin capsules and secured with a sinker to prevent them from floating. The dissolution media used were 0.1 mol·L^−1^ hydrochloric acid (pH ~1.2) and phosphate buffer (pH ~6.8). The paddles rotated at 50 rpm, and the temperature was kept at 37 °C. At designated intervals, a 2 mL sample was taken and replaced with pre-heated dissolution medium, then filtered through a 0.22-μm PTFE syringe filter. The dissolved amount of CUR was determined using a validated HPLC method.

### 3.10. In Vitro Parallel Artificial Membrane-Permeability Assay

A cell-free permeation model was used to assess intestinal permeability through passive diffusion. CUR was dissolved in dimethyl sulfoxide, while samples of physical mixtures and systems were prepared in the same manner as for the solubility test. Two 96-well plates were used for the experiment. A sample mixed with donor solution (pH 6.8) was added to each well of the acceptor plate, and each well of the donor plate was then filled with an acceptor sink buffer. GIT-0 lipid was deposited into the microfilter discs that divide the wells. The acceptor and donor plate were then joined and incubated for three hours at 37 °C. To analyze CUR concentrations, a validated HPLC method was utilized. The apparent permeability coefficient (*Papp*) was calculated using the following formulas:

$$Papp=\frac{-ln(1-\frac{C_{A}}{C_{equilibrium}})}{S\times \left(\frac{1}{V_{D}}+\frac{1}{V_{A}}\right)\times t}$$

$$C_{equilibrium}=\frac{C_{D}\times V_{D}+C_{A}\times V_{A}}{V_{D}+V_{A}}$$

where *C_A_* is the concentration in the acceptor well, *C_D_* is the concentration in the donor well, *V_D_* is the donor volume, *V_A_* is the acceptor volume, *S* is the membrane area, and *t* is the time (in seconds).

*Papp* values below 0.1 × 10^−6^ cm·s^−1^ are indicative of low-permeable substances; *Papp* values between 0.1 × 10^−6^ cm·s^−1^ and 1 × 10^−6^ cm·s^−1^ are indicative of medium-permeable substances; and *Papp* values greater than 1 × 10^−6^ cm·s^−1^ are indicative of highly permeable substances [57].

### 3.11. Biological Activity Assessment

#### 3.11.1. DPPH Assay

Antioxidant activity against the 2,2-diphenyl-1-picrylhydrazyl radical was evaluated using the previously published methodology [87], with minor modifications, as follows: 175 μL of a 0.2 mM DPPH solution was combined with 25 μL of the sample, and the mixture was shook for 30 min at room temperature in the dark. A UV–Vis microplate spectrophotometer (Multiskan GO, Thermo Fisher Scientific, Waltham, MA, USA) was used to measure absorbance at 517 nm. 175 μL of DPPH solution and 25 μL of distilled water were used as the control.

#### 3.11.2. Determination of Butyrylcholinesterase Inhibition

The inhibition of BChE activity was studied on the basis of the method of Stasiłowicz-Krzemień et al. [88] with minor modifications. Tests involved the use of 0.2 U·mL^−1^ of an enzyme, 1.5 mM of butyryltiocholine iodide (BTCI) as an artificial substrate, and 0.3 mM of 5,5′-dithio-bis-(2-nitrobenzoic) acid (DTNB). The reaction between DTNB and thiocholine, a product of BChE, produced the color development. Absorbance at 405 nm was measured using a UV–Vis microplate spectrophotometer (Multiskan GO, Thermo Fisher Scientific, Waltham, MA, USA). For inhibitory tests, samples were preincubated with BChE for 5 min before DTNB and BTCI were added. The inhibition of enzyme activity was calculated using the following equation:

$${BChE}_{inhibition\;}\left(\%\right)=\frac{1-\left(A_{1}-A_{1b}\right)}{\left(A_{0}-A_{0b}\right)}\times 100\%$$

where:

*A*_1_—the absorbance of the test sample.

*A*_1*b*_—the absorbance of the blank of the test sample.

*A*_0_—the absorbance of control.

*A*_0*b*_—the absorbance of the blank of control.

### 3.12. Statistical Analysis

The Statistica 13.3 program (StatSoft, Krakow, Poland) was used to conduct the statistical analysis. A one-way analysis of variance (ANOVA) was conducted on the collected data, followed by Duncan’s post hoc test. Statistical significance was established at a probability level of *p* < 0.05. Results are presented as mean ± standard deviation.

## 4. Conclusions

This study demonstrated the successful utilization of supercritical fluid technology in creating amorphous systems containing curcumin and a polymer, as well as a ternary system with curcumin, a polymer, and tryptophan. FT-IR spectroscopy indicated the formation of hydrogen bonds between curcumin and the polymer, curcumin and tryptophan, and the polymer and tryptophan, suggesting stabilization of the systems. This led to a significant enhancement in curcumin solubility, resulting in improved DPPH radical scavenging and butyrylcholinesterase inhibition capabilities.

This research showcases the potential of combining amorphization techniques with co-formers like tryptophan to significantly enhance the solubility, dissolution rate, and permeability of curcumin, addressing its limitations and potentially improving its therapeutic efficacy.

## Figures and Tables

**Table 1:** The results of solubility studies of CUR, physical mixtures and obtained amorphous systems.

CUR/Physical Mixture/Amorphous System	Concentration (µg·mL^−1^)
CUR	3.170 ± 0.000 ^c^
CUR:P(VP-co-VAc) ph. m.	3.300 ± 0.002 ^c^
CUR-P(VP-co-VAc)	781.600 ± 0.002 ^b^
CUR:TRP:P(VP-co-VAc) ph. m.	1.500 ± 0.001 ^c^
CUR-TRP-P(VP-co-VAc)	915.300 ± 0.004 ^a^

The statistically significant values are denoted by letters from ‘a’–‘c’, where ‘a’ indicates the highest value (*p* < 0.05).

**Table 2:** The results of pH measurements of CUR, TRP, physical mixtures and obtained amorphous systems.

Compound/Physical Mixture/Amorphous System	pH
CUR	5.14 ± 0.01
TRP	5.17 ± 0.02
P(VP-co-VAc)	4.46 ± 0.04
CUR:P(VP-co-VAc) ph. m.	4.28 ± 0.04
CUR-P(VP-co-VAc)	4.24 ± 0.03
CUR:TRP:P(VP-co-VAc) ph. m.	4.29 ± 0.01
CUR-TRP-P(VP-co-VAc)	4.34 ± 0.02

**Table 3:** The results of the biological activity tests regarding the ability to inhibit the DPPH radical and the BChE enzyme.

Compound/Physical Mixture/Amorphous System	Assay	
	DPPH	BChE
	% of Inhibition	% of Inhibition
CUR	13.38 ± 0.42 ^b^	2.97 ± 0.87 ^e^
TRP	12.15 ± 0.63 ^c^	0.51 ± 0.23 ^g^
P(VP-co-VAc)	5.89 ± 0.67 ^f^	2.07 ± 0.92 ^f^
CUR:P(VP-co-VAc) ph. m.	8.31 ± 0.58 ^e^	2.35 ± 0.63 ^d^
CUR-P(VP-co-VAc)	88.54 ± 1.51 ^a^	46.64 ± 0.74 ^b^
CUR:TRP:P(VP-co-VAc) ph. m.	9.79 ± 0.89 ^d^	4.55 ± 1.51 ^c^
CUR-TRP-P(VP-co-VAc)	88.85 ± 1.07 ^a^	74.54 ± 1.88 ^a^

The statistically significant values are denoted by letters from ‘a’–‘g’, where ‘a’ indicates the highest value (*p* < 0.05).

## Data Availability

Data are contained within the article.

## Supplementary Materials

The following supporting information can be downloaded at: https://www.mdpi.com/article/10.3390/ijms26020855/s1.
