# Supplementary material for: Curcumin Solubility and Bioactivity Enhancement Through Amorphization with Tryptophan via Supercritical Fluid Technology
Source: Int J Mol Sci. 2025 Jan 20;26(2):855. doi: 10.3390/ijms26020855 (PMC11766122; doi:10.3390/ijms26020855)
Supplement: Supplementary file 1 [file ijms-26-00855-s001.zip › ijms-3345171-supplementary.pdf]

Supplementary materials

Curcumin Solubility and Bioactivity Enhancement Through Amorphization with Tryptophan via Supercritical Fluid Technology

Ewa Garbiec, Natalia Rosiak, Szymon Sip, Przemysław Zalewski and Judyta Cielecka-Piontek *

Department of Pharmacognosy and Biomaterials, Poznan University of Medical Sciences, Rokietnicka 3 Str., 60-806 Poznan, Poland; ewa.garbiec@student.ump.edu.pl (E.G.); nrosiak@ump.edu.pl (N.R.); szymonsip@ump.edu.pl (S.S.); pzalewski@ump.edu.pl (P.Z.)

* Correspondence: jpiontek@ump.edu.pl

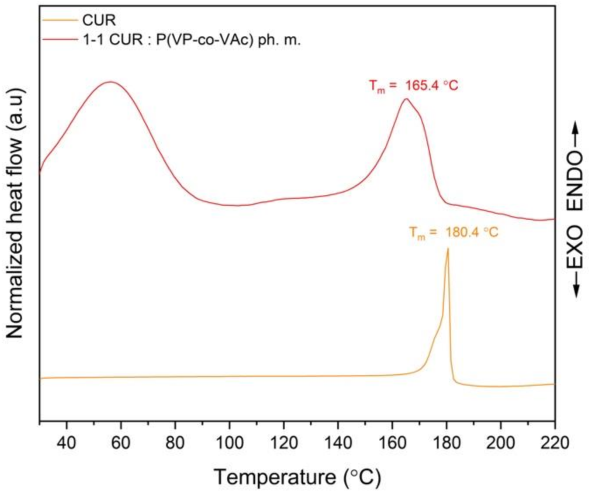

Figure S1. DSC thermograms of CUR and CUR:P(VP-co-VAc) physical mixture prepared at a 1:1 ratio.

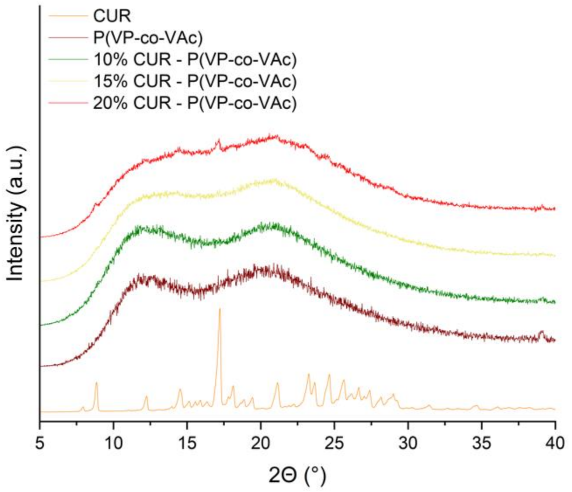

Figure S2. XRPD diffraction patterns of CUR, P(VP-co-VAc), and binary CUR-P(VP-co-VAc) system with 10%, 15%, and 20% CUR content.

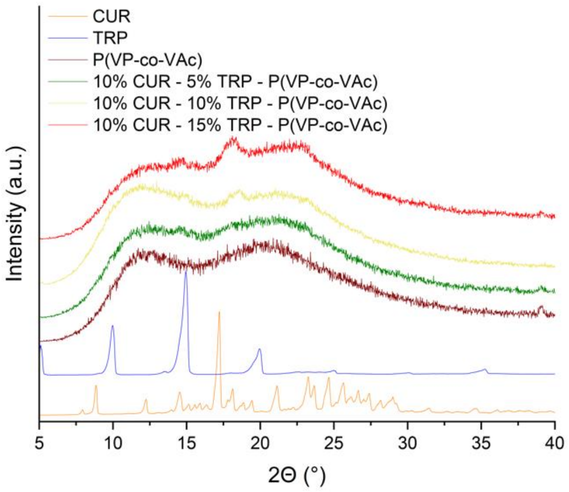

Figure S3. XRPD diffraction patterns of CUR, TRP, P(VP-co-VAc), and ternary CUR-TRP-P(VP-co-VAc) system with 10% CUR and 5%, 10%, and 15% TRP content.

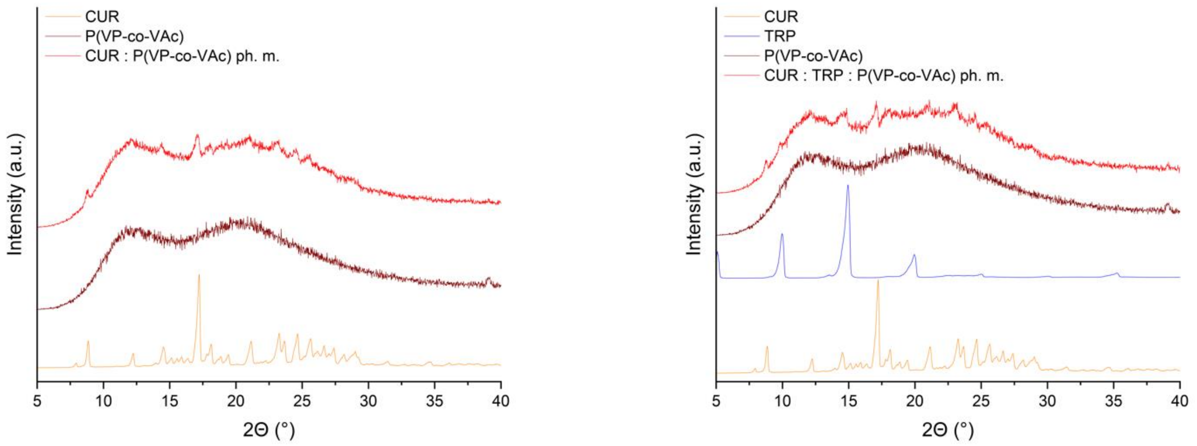

(a) (b)

Figure S4. XRPD diffraction patterns of CUR, P(VP-co-VAc), and binary CUR:P(VP-co-VAc) physical mixture **(a)** and CUR, TRP, P(VP-co-VAc), and ternary CUR:TRP:P(VP-co-VAc) physical mixture **(b)**.

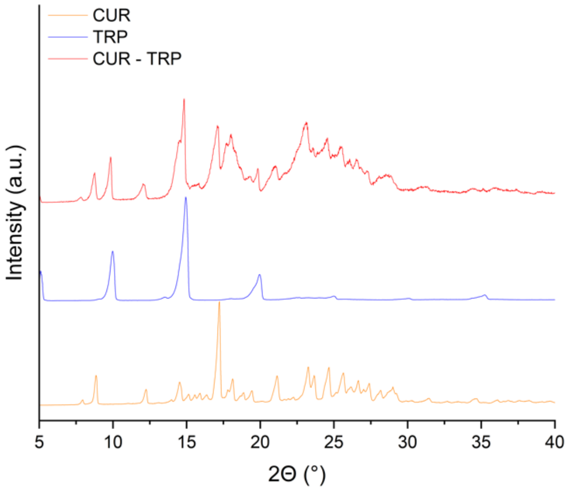

Figure S5. XRPD diffraction patterns of CUR, TRP, and CUR-TRP system prepared by means of a SCF method.

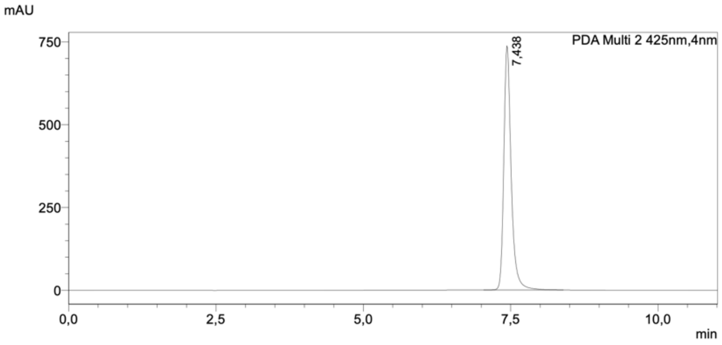

Figure S6. Chromatogram of CUR for the developed method.

Table S1. Validation parameters of HPLC-DAD method for concentration determination of CUR.

Linearity range (mg·mL^{-1})	0.00002-0.3
Correlation coefficient (r)	0.9998
a ± Sa	11149771288.40 ± 43899858.98
b ± Sb	insignificant (α=0.05)
LOD (mg·mL^{-1})	0.00003899
LOQ (mg·mL^{-1})	0.00011815
Retention time (min)	7.44
